# Can Pure Silk Compete with the Established Mepilex Ag^®^ in the Treatment of Superficial Partial Thickness Burn Wounds? A Prospective Intraindividual Study

**DOI:** 10.3390/ebj6030041

**Published:** 2025-07-11

**Authors:** Jan Akkan, Mahsa Bagheri, Sophia Mezger, Paul Christian Fuchs, Maria von Kohout, Wolfram Heitzmann, Rolf Lefering, Jennifer Lynn Schiefer

**Affiliations:** 1 Department of Plastic, Reconstructive, Hand and Burn Surgery, Hospital Cologne Merheim, University of Witten-Herdecke, 51109 Cologne, Germanysophia-mezger@hotmail.de (S.M.); fuchsp@kliniken-koeln.de (P.C.F.); vonkohoutm@kliniken-koeln.de (M.v.K.); heitzmannw@kliniken-koeln.de (W.H.); schieferj@kliniken-koeln.de (J.L.S.); 2 Department of Plastic and Aesthetic Surgery, Emil von Behring Hospital, 14165 Berlin, Germany; 3 Institute for Research in Operative Medicine, Hospital Cologne Merheim, University of Witten-Herdecke, 51109 Cologne, Germany; rolf.lefering@uni-wh.de

**Keywords:** wound dressings, burns, superficial partial thickness, pure silk, Mepilex Ag^®^

## Abstract

Introduction: Superficial partial thickness burns generally do not require surgical intervention and are managed with specialized wound dressings. Mepilex Ag^®^ is commonly used and often represents the standard of care. This study evaluated the clinical performance of pure silk compared to Mepilex Ag^®^. Methods: A prospective, single-center intraindividual study was conducted on adult patients with superficial partial thickness burns. Each burn wound was divided, treating one half with pure silk and the other with Mepilex Ag^®^. Clinical parameters including wound closure time, pain levels, and scar quality at 3-month follow-up were analyzed. Results: Twenty-four patients were included (mean TBSA: 5.8%). Mepilex Ag^®^ showed a trend towards a shorter wound closure time (10.5 vs. 11.5 days; *p* = 0.223). Pain scores remained below 4/10 for both dressings throughout treatment. However, Mepilex Ag^®^ demonstrated significantly lower pain on day one (3.5 vs. 2.77; *p* = 0.039) and day two (2.91 vs. 2.27; *p* = 0.041). Scar quality after 3 months was similar. Conclusion: Both dressings proved to be effective treatment options. Pure silk required fewer resources, showed high clinical practicality, and demonstrated a similar performance to Mepilex Ag^®^ in key clinical parameters, making it an interesting option for other clinics and our standard of care.

## 1. Introduction

Burn injury is globally recognized as the fourth most prevalent form of trauma, resulting in an estimated 180,000 fatalities annually, according to the World Health Organization (WHO) [1]. The management of burn injuries presents multiple distinct challenges that often demand a specialized therapeutic approach [2]. A major challenge lies in the adequate treatment of the burn wound which is highly dependent on burn wound depth [3]. Burn wounds classified as deep partial thickness or full thickness typically require surgical necrectomy followed by subsequent skin grafting [3]. In contrast, superficial partial thickness burn wounds typically do not require surgical intervention and are frequently managed with specialized burn wound dressings [4].

During the second half of the 20th century, a diverse array of biological and synthetic dressings for burn wounds was developed and subsequently integrated into clinical practice [5,6]. Several essential attributes of an effective burn wound dressing have been identified, including: (1) creation of an optimal environment for wound healing, (2) reduction of pain, (3) prevention of water loss, (4) provision of a bacterial barrier, and (5) promotion of good scar quality [5,6,7]. Despite substantial research focused on the critical evaluation and comparison of different synthetic and biological burn wound dressings, a perfect dressing has not yet been developed [8].

Specialized burn wound dressings regularly used for the definite treatment of superficial partial thickness burn wounds include silver-containing dressings such as Mepilex Ag^®^ [9,10]. Recently, dressings based on pure silk have also been studied in the context of burn wounds [11]. Dressilk^®^ (Prevor, Valmondois, France) is a biological wound dressing that consists of the silk fibroin (SF) protein produced by silk worms [12]. Silk-based wound dressings have been shown to promote wound healing, while also offering advantages in terms of cost-efficiency and ease of use in the context of burn wounds [13,14]. Mepilex Ag^®^ (Moelnlycke Heath Care, Goeteborg, Sweden) is a synthetic wound dressing that consists of a soft silicone wound contact layer, an absorbent foam pad containing silver sulfate and a vapor-permeable superficial layer [15]. Mepilex Ag^®^ displays high antimicrobial activity and was shown to be an effective dressing in burn patients in terms of patient comfort [9,16].

The objective of this study was to perform a prospective intraindividual comparison of Dressilk^®^ and Mepilex Ag^®^ wound dressings in superficial partial thickness burn wounds in terms of patient comfort, wound healing, exudation, infection and scarring. While both dressings have been previously studied within the context of burn wounds, to our knowledge, no direct comparisons have been made up to this point. A comprehensive comparative analysis between Dressilk^®^ and Mepilex Ag^®^ could serve to delineate the advantages of the respective dressings, thereby assisting medical professionals in choosing the appropriate option from both clinical and economic perspectives.

## 2. Materials and Methods

### 2.1. Ethics

This study was evaluated by the ethics committee of the University of Witten-Herdecke with approval granted on 16 December 2019. The study was designed in accordance with the ethical principles of scientific research of the updated declaration of Helsinki of 2013.

### 2.2. Patient Group

All patients between the age of 17 and 80 years that presented with burn injuries to the burn center of the Merheim Hospital in Cologne from 2020 to 2022 were considered for inclusion in the study. Additional inclusion criteria comprised a total body surface area (TBSA) of at least 0.5% and burn wound depth classified as exclusively superficial partial thickness. Furthermore, only patients who were selected for conservative therapy without surgical necrectomy as the standard of care (SOC) were included in this study. All patients that presented with burn injuries to the face or were suspected to have sustained an inhalation trauma were excluded from this study. Further exclusion criteria involved the presence of an active infection, a history of steroid medication, and patients that were pregnant or breastfeeding. Patients that failed to complete the follow-up examinations were also excluded from this study.

### 2.3. Study Design

The study was designed as a prospective, single-center, intraindividual clinical study. Primary outcome of this study was the time to wound closure defined as less than 5% of the initial wound area remaining. The evaluation was performed clinically by the principal investigator using visual inspection and standardized photography. Wound size was estimated by comparing the remaining open wound area to the initial wound surface, based on clinical measurement. The dressings and burn wounds were critically examined, and segments covering wound areas that had already epithelialized were carefully removed to enable an accurate assessment of wound closure. The time point at which 95% of the wound was re-epithelized was noted for both wound dressings. Secondary outcomes were infection, bleeding, burn wound secretion, pain, necessity of burn wound dressing changes and scar quality after 3 months using the Vancouver Scar Scale (VSS). Pain intensity was quantified using the Numeric Rating Scale (NRS) (0 = no pain, 10 = maximum pain) on days 1, 2, 4, 8, 12, 16, and 24 post-therapy initiation. Pain intensity was assessed during the change of the superficial dressing on the specified days for both wound dressings. Pain associated with the change of the primary dressing, which occurred every four days in the case of Mepilex Ag^®^, was not included in the statistical analysis to maintain comparability between the two dressing types. The wound secretion was assessed by the attending burn surgeon on days 1, 2, 4, 8, 12, 16, and 24 after the beginning of therapy using the visual rating scale (VRS) (0 = no exudation, 10 = maximum exudation). The level of exudation was evaluated by the principal investigator. Exudation was assessed by the appearance and the level of moisture of the specialized burn wound dressing itself and the superficial dressings on top. The VSS was first described in 1990 and aims to quantify scar quality based on pigmentation, vascularization, pliability and height [17]. All scar evaluations were performed by the same examiner.

### 2.4. Study Process

All patients that met the criteria mentioned above were comprehensively informed about the study, including its associated risks, and provided written informed consent prior to participation. The initial intervention involved debridement of the burn wound using Prontosan^®^ solution and compresses as per SOC. The burn depth was assessed by the attending burn surgeon and afterwards the wound was photographed. Patients presenting with partial superficial burn wounds were included in the study after which the wound was divided into two parts of equal size. One half of the burn wound was covered with Dressilk^®^ wound dressing, while the other half of the wound received Mepilex Ag^®^ wound dressing upon the day of admission. The wound areas treated with the respective burn wound dressings were chosen randomly. Subsequently, a superficial protective dressing with fatty gauze and compresses was applied and patients were admitted to the burn department. A first superficial dressing change was carried out on the first day after admission.

The Mepilex Ag^®^ wound dressings were changed every 4 days in accordance with the manufacturer’s guidelines, which recommend use for “several days,” as the antimicrobial effect was shown to constantly decrease and fully diminish after 7 days [18]. A new Dressilk^®^ wound dressing was only applied if the old dressing detached from the wound. The treatment with respective burn wound dressings was continued until re-epithelization of the wound occurred and the dressings naturally detached. Superficial dressing changes using fatty gauze and sterile non-adherent dressings were performed on days 1 and 2, and subsequently every other day until discharge from the hospital. This approach was particularly important in the case of Mepilex Ag^®^, as the primary dressing required additional fixation using a superficial bandage. In contrast, the Dressilk^®^ dressing was left exposed once wound exudation had diminished and sufficient adherence to the wound bed was achieved. Fatty gauze was used for both dressings during the initial days of treatment with high levels of exudation to avoid sticking of the specialized burn wound dressing to the secondary dressings. In the case of Mepilex Ag^®^, although the use of additional layers is not typically described in the manufacturer’s protocol, fatty gauze was used to reduce adherence to secondary dressings and to facilitate atraumatic dressing changes during the initial days of treatment. Following discharge, the monitoring of the burn wound and the burn wound dressings was carried out in the outpatient clinic until wound closure was achieved (defined as epithelization of >95% of the initial wound area). Patients were seen in the outpatient clinic every 2 days to allow for a thorough monitoring of clinical parameters. A routine follow-up appointment was scheduled 3 months post-therapy initiation to evaluate scar quality using the VSS.

### 2.5. Statistics

The statistical analysis was performed using SPSS software (Version 22.0, IBM Inc., Armonk, NY, USA). A *p*-value of <0.05 was set as a cutoff for statistical significance. Analysis of the data was performed using descriptive statistics, the paired *t*-test for the clinical data (infection, bleeding, burn wound secretion, pain, dressing changes), and the Wilcoxon Signed-Rank test for the comparison of the VSS scores.

## 3. Results

**Figure 1:**
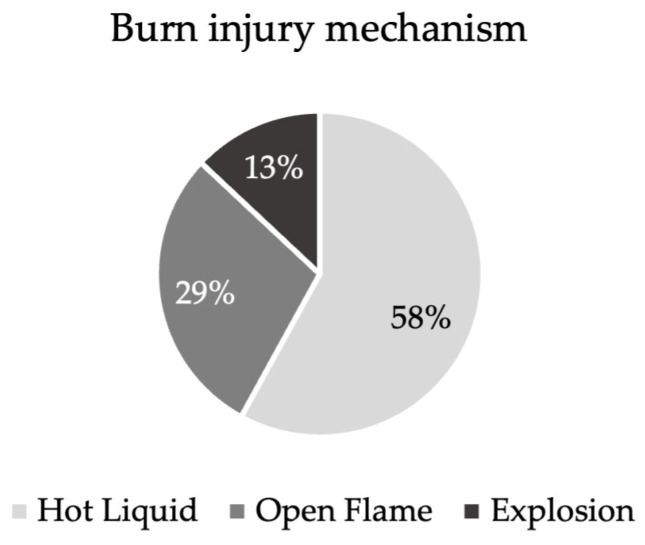
Relative distribution of burn mechanisms in the patient cohort (n = 24). The diagram depicts the relative number of patients injured by the different burn mechanisms as a percentage: hot liquid (58%), open flame (29%), and explosion (13%).

A total of 24 patients presenting with partial superficial burn wounds to the burn center of the Merheim Hospital between 2020 and 2022 were included in this prospective study. The group comprised 13 male patients and 11 female patients, with ages ranging from 17 to 79 years and a mean age of 39 years. The majority of burns were caused by hot liquids (58%; 14/24 patients), followed by open flames (29%; 7/24 patients), and explosions (13%; 3/24 patients) (Figure 1). Injury mechanisms included scalds caused by hot water bottles, flame injuries resulting from kitchen accidents, and explosion-related burns due to fireworks or malfunctioning lighters. The most common locations for burn wounds were the legs (37.5%), the arms (25%), and the hands (25%), with less frequent occurrences on the feet, back, and chest (4.16% each). The mean TBSA affected was 5.8%, with individual cases ranging from 1% to 14.5%.

### 3.1. Wound Healing

**Figure 2:**
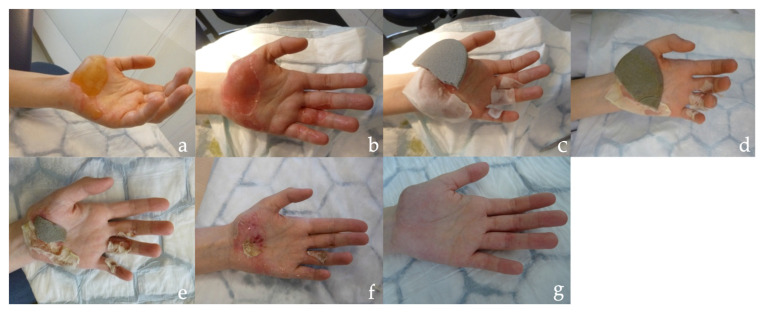
Wound healing of a patient who sustained a partial superficial burn to the left hand. Half of the burn wound at the palm was treated with Dressilk^®^ and the other half of the wound was treated with Mepilex Ag^®^. Photos show the wound at different time points of the therapy: (**a**) initial presentation on the day of the trauma, (**b**) post-debridement, (**c**) after application of Dressilk^®^ on the ulnar side and Mepilex Ag^®^ on the radial side of the wound (the Mepilex Ag^®^ is partially detached at the edges), (**d**) clinical appearance after four days, (**e**) clinical appearance after eight days, (**f**) clinical appearance after four weeks, (**g**) clinical appearance after three months.

The two dressings, Dressilk^®^ and Mepilex Ag^®^, were applied to the wounds and treatment was continued until the dressings naturally detached following re-epithelization. The Mepilex Ag^®^ wound dressings were changed every four days and were gradually reduced in size as re-epithelization of the wound progressed. Throughout the healing process, both dressings progressively dried out and became increasingly rigid (Figure 2). The natural elasticity of silk allowed for precise modulation of the Dressilk^®^ wound dressings in body areas with rounded surfaces such as the arms, the legs and the hands. After removing the protective film, the Mepilex Ag^®^ wound dressing adhered to the burn wound but needed to be secured with a superficial bandage in areas with pronounced curves, such as the hand (Figure 2c). The Dressilk^®^ wound dressing became semitransparent once applied to the wound and consequently allowed for a continuous inspection and monitoring of the wound. The Mepilex Ag^®^ remained in its initial non-transparent form until detachment. No infections or bleeding were observed in any of the patients within the study group. In all patients, the initially applied Dressilk^®^ wound dressing remained attached on the burn wound until re-epithelization. The Mepilex Ag^®^ wound dressings were changed regularly every 4 days and maintained secure adhesion in most patients. In some patients, the Mepilex Ag^®^ wound dressings partially detached in body areas with pronounced curves, necessitating the use of a superficial bandage, which was regularly changed for proper fixation. In most cases, the Dressilk wound dressing could be left exposed from the second day after application, eliminating the need for further bandage changes.

### 3.2. Pain

**Figure 3:**
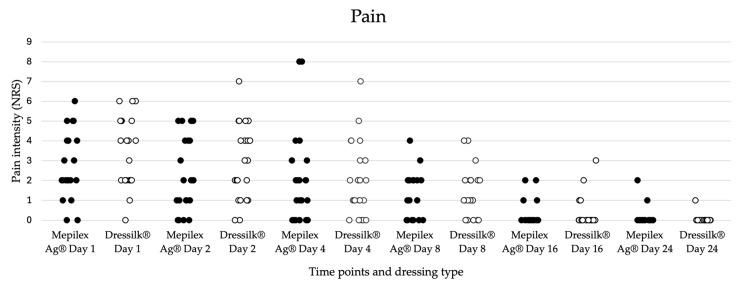
Dot plot diagram of pain levels as reported by the patients (n = 22) using the Numeric Rating Scale (NRS). Comparison of the wound area treated with Mepilex Ag^®^ (represented in black) and Dressilk^®^ (represented in white) at different time points (days 1, 2, 4, 8, 16, 24). Reported pain levels in areas treated with Mepilex Ag^®^ were significantly lower on day 1 (*p* = 0.039) and day 2 (*p* = 0.041). Statistical significance was set at *p* < 0.05.

Initial mean pain levels, as reported by the patients using the Numeric Rating Scale (NRS) were 2.77 for Mepilex Ag^®^ and 3.5 for Dressilk^®^, and consistently decreased during the wound healing process (Table 1, Figure 3). The pain level data was complete in 22 patients and was consequently included in the statistical analysis. Pain levels in the area treated with Dressilk^®^ were significantly higher on day 1 (3.5 vs. 2.77; *p* = 0.039) and day 2 (2.91 vs. 2.27; *p* = 0.041) after application of the dressings. In the subsequent days, no significant differences in pain levels were observed and pain levels gradually decreased, eventually reaching 0 at day 24 for both dressings.

### 3.3. Exudation

**Figure 4:**
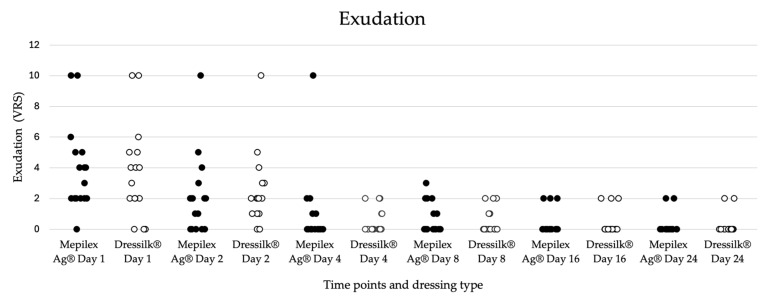
Dot plot diagram of the level of exudation in the patient group (n = 18) as evaluated by the attending burn surgeon using the visual rating scale (VRS). Comparison of the wound area treated with Mepilex Ag^®^ (represented in black) and Dressilk^®^ (represented in white) at different time points (days 1, 2, 4, 8, 16, 24). There was no significant difference between the two wound dressings. Statistical significance was set at *p* < 0.05.

The level of exudation was evaluated by the attending burn surgeon using the Visual Rating Scale (VRS), with initial scores of 3.78 for Mepilex Ag^®^ and 3.61 for Dressilk^®^ on day 1 (Table 1, Figure 4). The exudation level data was complete in 18 patients and was consequently included in the statistical analysis. The levels of exudation constantly decreased, reaching 0.22 on day 24 for both wound dressings. No statistical difference was observed at any time point during the wound healing process. While the Mepilex Ag^®^ dressing tended to absorb the majority of wound exudate, the Dressilk^®^ dressing allowed most of the exudate to pass through to the secondary dressing layer.

### 3.4. Time to Wound Closure

The mean time to wound closure was 10.5 days (standard deviation (SD): 4.95) for the wound areas treated with Mepilex Ag^®^ and 11.5 days (SD: 6.56) for those treated with Dressilk^®^. Data on the time to wound closure was complete in 22 patients and was consequently included in further analysis. The time to wound closure for areas treated with Mepilex Ag^®^ ranged from 4 to 21 days, whereas for Dressilk^®^-treated areas, it ranged from 4 to 28 days. Although the mean time to wound closure was shorter for Mepilex Ag^®^-treated areas (10.5 days vs. 11.5 days), this difference was not statistically significant (*p* = 0.223).

### 3.5. Infection and Bleeding

None of the patients included in this study showed clinical signs of infection or bleeding during the 3-month follow-up.

### 3.6. Scar Quality

The scar quality assessed by the VSS at 3 months after initiation of therapy showed no significant difference in terms of vascularization, pigmentation, pliability, height or overall VSS score between the respective burn wound dressings. A trend towards a lower VSS score and consecutively high scar quality was seen in areas treated with Mepilex Ag^®^ compared to areas treated with Dressilk^®^ (mean VSS score: 2.9 vs. 4.1; *p* = 0.087).

### 3.7. Loss to Follow-Up

Four patients (7%) were excluded from this study due to failure to complete the follow-up. As a result, the number of patients was reduced from an initial 28 to 24. In all four cases, the reason for loss to follow-up was the long travel distance to the hospital.

## 4. Discussion

This is the first prospective intraindividual study comparing Dressilk^®^ to the widely established Mepilex Ag^®^ wound dressing in superficial partial thickness burn wounds. Although the fundamental therapeutic principles in the management of burn wounds are well-established, the search for the optimal burn wound dressing remains a topic of ongoing debate and active research. Mepilex Ag^®^ has been thoroughly studied and compared to other burn wound dressings in the context of partial thickness burns by multiple previous studies. In contrast, comparative studies on Dressilk^®^ in burn wounds are limited. Even though a perfect dressing has not yet been developed, the specific requirements have been defined by previous studies: (1) creation of an optimal environment for wound healing, (2) patient comfort, (3) prevention of water loss, (4) provision of a bacterial barrier, (5) promotion of good scar quality, (6) low cost, (7) availability, (8) easy storage, (9) durability and (10) adaptation to surfaces [5,6]. In this study, we demonstrated that both wound dressings, Dressilk^®^ and Mepilex Ag^®^, are effective options for treating superficial partial thickness burns and meet many of the aforementioned requirements.

Both wound dressings, Dressilk^®^ and Mepilex Ag^®^, were easily applicable to the burn wound and enabled an effective wound coverage until re-epithelialization was complete. In concordance, Dressilk^®^ has previously been described as a stable material, shown to reliably adhere to burn wounds and be easy to use [12,14]. Similarly, multiple studies have reported that Mepilex Ag^®^ is easier to handle compared to other burn wound dressings [9,19]. In our clinical experience, Dressilk^®^ wound dressings demonstrated higher user-friendliness due to the material’s properties, which made modulation to the body surface significantly easier. The thin and semitransparent structure of the Dressilk^®^ dressing allowed for a more accurate assessment of the burn wound. Additionally, once dried, the Dressilk^®^ wound dressings remained securely fixed to the wound bed, generally eliminating the need for additional bandages. In this study, the Mepilex Ag^®^ wound dressings exhibited partial detachment at the edges of the dressing in some cases in body areas with pronounced curves, due to their comparatively more rigid and thicker structure. This required the fixation of the wound dressings with superficial bandages, demanding increased resources in terms of materials and personnel. In addition, the treatment with Mepilex Ag^®^ necessitated regular changes of the wound dressing itself as the manufacturer recommends a therapy duration of several days, with the antimicrobial effect ceasing after 7 days. In our clinic, Mepilex Ag^®^ burn wound dressings were changed every 4 days. This is established as a standard in our clinic to comply with the manufacturer’s recommendations and ensure a consistently high antimicrobial effect. This further demands personnel resources and potentially causes pain for the patient due to the direct manipulation of the wound surface. Regarding wound exudation, no significant difference was observed between the two wound dressings during the follow-up. Level of exudation was generally low, which is in line with previous studies of Dressilk^®^ and Mepilex Ag^®^ in partial thickness burn wounds [11,20].

Pain is a characteristic feature of superficial partial thickness burn wounds and thus represents a major challenge in the management of burn patients [21,22]. In our study, reported pain levels were significantly lower in burn areas treated with Mepilex Ag^®^ during the first two days of treatment. Overall, patients reported low mean pain levels on the NRS for both burn wound dressings during the early days of treatment, with mean pain levels reaching 2 for both dressings by day 4. Studies comparing pain levels of patients with partial thickness burn wounds treated with Mepilex Ag^®^ to other established burn wound dressings (e.g., Biobrane^®^ and Acticoat^®^) reported no significant differences [19,23]. In contrast, Hundeshagen et al. showed pain levels to be significantly lower in patients treated with the more expensive Suprathel^®^ burn wound dressing [9].

Burn wound infection presents a major life-threatening complication and therefore rapid achievement of burn wound closure is essential [24]. In our study, none of the patients showed clinical signs of burn wound infection. As TBSA has been identified as a major risk factor for infection, the relatively small mean TBSA in our patient cohort could be a possible explanation [25]. In addition, Mepilex Ag^®^ has been shown to effectively reduce bacterial load compared to other established burn wound dressings in a laboratory setting by Szweda et al. [16]. This is particularly relevant as the authors conducted the study with types of bacteria that have been shown to play a major role in burn wound infections including *Staph. aureus*, *Pseudomonas aeruginosa* and *E. coli* [16,24]. In terms of time to wound closure, wound areas covered with Mepilex Ag^®^ showed a tendency to heal faster compared to areas treated with Dressilk^®^ (10.5 vs. 11.5 days), although the difference was not statistically significant. Overall, these findings are consistent with the time to wound closure reported by previous studies of Dressilk^®^ and Mepilex Ag^®^ dressings in partial thickness burn wounds [9,14].

The development of scars is a common complication after burn trauma and can potentially lead to severe impairments of the patient’s quality of life [26]. In the 3-month follow-up examination in this study, we could observe no significant difference in scar quality between Dressilk^®^ and Mepilex Ag^®^ in the VSS. Previous studies have reported treatment with Mepilex Ag^®^ to result in satisfactory scar quality in comparison with other burn wound dressings [9,19]. Aggarwala et al. reported a mean VSS score of 1.1 in burn wounds treated with Mepilex Ag^®^ in the 3-month follow-up, which was significantly lower than the VSS score in wounds treated with Biobrane^®^ or Aquacel Ag^®^ [19]. Interestingly, Schiefer et al. observed a significantly lower vascularization of scars in wounds treated with Dressilk^®^ compared to Suprathel^®^ [11]. In summary, both wound dressings, Dressilk^®^ and Mepilex Ag^®^, seem to result in satisfactory scar quality according to our study and the previous literature.

The management of burn patients presents a high financial burden, and therefore low cost is an essential requirement for a burn wound dressing as previously described by Pruitt et al. and Tsai et al. [5,27]. Dressilk^®^ has been identified as a cost-effective burn wound dressing by previous studies in comparison to Suprathel^®^ and Biobrane^®^ [11,28]. Interestingly, Mepilex Ag^®^ has also been shown to have economic advantages in comparison to Suprathel^®^ and Biobrane^®^ as well as other established burn wound dressings including Acticoat^®^ [9,19,29]. In our clinic, the cost of Mepilex Ag^®^ is approximately 40% of that of Dressilk^®^ wound dressings, making it the more cost-effective option in terms of initial acquisition. However, the Mepilex Ag^®^ wound dressing required regular changes over the course of the treatment and needed to be fixed with superficial bandages in a subset of patients. Changing the wound dressing every 4 days over the course of an average treatment duration of 10.5 days often balanced out the initially lower acquisition cost. In addition, the need for more frequent changes of the Mepilex Ag^®^ dressings and superficial bandages required increased personnel resources. A longer interval between dressing changes of Mepilex Ag^®^ (e.g., 5–7 days) may contribute to a reduction in treatment costs and will be re-evaluated in future clinical practice at our institution. In addition, as fatty gauze is not typically included in the manufacturer’s protocol, using the Mepilex Ag^®^ dressing without it could further reduce overall treatment costs. Overall, both wound dressings resulted in comparable overall treatment costs in our study.

### Limitations

This study has several limitations. The study group of 24 patients included in this study is relatively small and therefore reduces statistical power. In addition, some patients (7%) were lost during follow-up examinations and consequently excluded from the study. A possible explanation is that patients included in this study had a mean TBSA of 5.8%. In our clinic, these patients are typically discharged after 2 to 3 days, which renders a thorough daily clinical documentation difficult. Furthermore, superficial partial thickness burn wounds typically heal within 2 to 3 weeks, which may reduce patients’ motivation to routinely return to the clinic for follow-up examinations.

Moreover, the intraindividual design of this study could possibly impede accurate subjective pain assessment. In patients with small burn wound areas, the burn wound dressings had to be placed in close proximity, which might hinder accurate differentiation by the patient. To mitigate this effect, the respective burn wound dressings were placed far apart from each other whenever possible.

In addition, since wound healing time is a key parameter of this study, the healing process should have been assessed and presented in greater detail. Providing exact percentages of wound closure for both dressings at each time point in every patient would have significantly improved the clarity and interpretability of the results. In future studies, we will adapt our protocol to include precise, quantitative documentation of wound closure at regular intervals.

Furthermore, a 3-month follow-up is an early time point to evaluate scar quality as the scar is subject to further changes. A longer follow-up period is required to allow for a thorough scar quality comparison.

Lastly, blinding of the medical personnel and study staff was not possible and therefore may have introduced bias into the study.

## 5. Conclusions

Dressilk^®^ and Mepilex Ag^®^ are effective and safe wound dressings in superficial partial thickness burn wounds according to our study. Both dressings enabled effective burn wound management with no complications and satisfactory scar quality after three months. Overall, both dressings resulted in comparable healing times, clinical outcomes, and treatment costs. Mepilex Ag^®^ appears to offer advantages in terms of lower pain levels during the initial days of treatment and lower acquisition costs. In our study, Dressilk^®^ demonstrated advantages in wound assessment after application and clinical user-friendliness. Therefore, Dressilk^®^ has been established as the standard of care (SOC) for superficial partial thickness burns in our clinic.

## Figures and Tables

**Table 1:** Comparison of the pain and exudation levels between Mepilex Ag^®^ and Dressilk^®^ on different time points. All values are presented as the mean value with the standard deviation in brackets. A significant difference of pain levels between Mepilex Ag^®^ and Dressilk^®^ was observed on day 1 (*p* = 0.039) and day 2 (*p* = 0.041). * Statistical significance was set at *p* < 0.05.

Parameter	n	Time	Mepilex Ag^®^	Dressilk^®^	*p*-Value
Pain	22	Day 1	2.77 (1.66)	3.50 (1.77)	0.039 *
		Day 2	2.27 (1.80)	2.91 (1.88)	0.041 *
		Day 4	2.00 (2.33)	2.00 (1.88)	0.730
		Day 8	1.27 (1.12)	1.36 (1.22)	0.577
		Day 16	0.27 (0.63)	0.32 (0.78)	0.655
		Day 24	0.14 (0.47)	0.05 (0.21)	0.317
Exudation	18	Day 1	3.78 (2.69)	3.61 (2.91)	0.450
		Day 2	2.11 (2.52)	2.50 (2.26)	0.174
		Day 4	1.44 (3.19)	1.00 (2.38)	0.655
		Day 8	0.61 (0.98)	0.44 (0.78)	0.317
		Day 16	0.33 (0.77)	0.33 (0.77)	1.0
		Day 24	0.22 (0.65)	0.22 (0.65)	1.0

## Data Availability

The original contributions presented in this study are included in the article material. Further inquiries can be directed to the corresponding author.
